# Reliability of System Identification Techniques to Assess Standing Balance in Healthy Elderly

**DOI:** 10.1371/journal.pone.0151012

**Published:** 2016-03-08

**Authors:** Jantsje H. Pasma, Denise Engelhart, Andrea B. Maier, Ronald G. K. M. Aarts, Joop M. A. van Gerven, J. Hans Arendzen, Alfred C. Schouten, Carel G. M. Meskers, Herman van der Kooij

**Affiliations:** 1 Department of Rehabilitation Medicine, Leiden University Medical Center, Leiden, the Netherlands; 2 Department of Biomechanical Engineering, Delft University of Technology, Delft, the Netherlands; 3 Department of Biomechanical Engineering, Institute for Biomedical Technology and Technical Medicine (MIRA), University of Twente, Enschede, the Netherlands; 4 Department of Medicine and Aged Care, Royal Melbourne Hospital, University of Melbourne, Melbourne, Australia; 5 Department of Human Movement Sciences, MOVE Research Institute Amsterdam, Vrije Universiteit Amsterdam, Amsterdam, The Netherlands; 6 Department of Mechanical Automation and Mechatronics, University of Twente, Enschede, the Netherlands; 7 Center for Human Drug Research, Leiden, the Netherlands; 8 Department of Rehabilitation Medicine, VU University Medical Center, Amsterdam, the Netherlands; University of Tuebingen, GERMANY

## Abstract

**Objectives:**

System identification techniques have the potential to assess the contribution of the underlying systems involved in standing balance by applying well-known disturbances. We investigated the reliability of standing balance parameters obtained with multivariate closed loop system identification techniques.

**Methods:**

In twelve healthy elderly balance tests were performed twice a day during three days. Body sway was measured during two minutes of standing with eyes closed and the Balance test Room (BalRoom) was used to apply four disturbances simultaneously: two sensory disturbances, to the proprioceptive and the visual system, and two mechanical disturbances applied at the leg and trunk segment. Using system identification techniques, sensitivity functions of the sensory disturbances and the neuromuscular controller were estimated. Based on the generalizability theory (G theory), systematic errors and sources of variability were assessed using linear mixed models and reliability was assessed by computing indexes of dependability (ID), standard error of measurement (SEM) and minimal detectable change (MDC).

**Results:**

A systematic error was found between the first and second trial in the sensitivity functions. No systematic error was found in the neuromuscular controller and body sway. The reliability of 15 of 25 parameters and body sway were moderate to excellent when the results of two trials on three days were averaged. To reach an excellent reliability on one day in 7 out of 25 parameters, it was predicted that at least seven trials must be averaged.

**Conclusion:**

This study shows that system identification techniques are a promising method to assess the underlying systems involved in standing balance in elderly. However, most of the parameters do not appear to be reliable unless a large number of trials are collected across multiple days. To reach an excellent reliability in one third of the parameters, a training session for participants is needed and at least seven trials of two minutes must be performed on one day.

## Introduction

Impaired standing balance is a significant problem in elderly [1;2] and is one of the main risk factors and causes of falling [3;4]. Falls often result in serious injuries, including death [5]. In standing balance, several underlying systems (i.e. muscles, neural system and sensory systems) interact, which results in a closed loop system in which cause and effect are interrelated [6]. The underlying systems deteriorate with age and are influenced by diseases and medication use [7–10]. Due to redundancy, these systems can compensate for each other’s deterioration. Therefore, the underlying cause of impaired standing balance is difficult to detect and hence, to intervene with targeted therapies [11].

Current clinical balance tests, such as posturography, do not take aforementioned cause and effect relations and redundancy of standing balance into account and therefore cannot detect the underlying cause of impaired standing balance [11]. Previous research showed that system identification techniques are useful to assess the underlying systems of standing balance, in which the response to well-known disturbances are assessed [12–16]. A clear advantage is that this method takes into account the cause and effect relation and separates the contribution of the underlying systems. This gives the opportunity to improve diagnosis of impaired balance and, eventually, to prevent falling by targeted therapies [6]. Before introducing the method into clinical practice for diagnosing or monitoring treatment of impaired balance, it is important to assess the reliability of this technique, which is yet unknown, and compare it with posturography.

In this study we investigated the reliability of standing balance parameters obtained with four disturbances applied simultaneously and system identification techniques to assess standing balance in healthy elderly and compared this with a parameter obtained with posturography, namely body sway. We used the generalizability theory (G theory) [17], which takes into account both systematic and random measurement errors. A validity study was performed to assess whether differences in standing balance parameters could be detected as expected by the results of previous studies, in which sensory reweighting was investigated by increasing disturbance amplitudes over trials using the same system identification techniques [12;15;18]. Furthermore, recommendations will be given for study designs to reduce the measurement errors and therefore improve the reliability.

## Materials and Methods

### Participants

Twelve healthy elderly aged 70 years or older participated in this study. Participants were recruited from the database of the Center of Human Drug Research, Leiden, the Netherlands, and the MyoAge study database of the Leiden University Medical Center, Leiden, the Netherlands. Participants were screened before entry to the study. Participants were excluded in case of low cognitive function (Mini Mental State Examination (MMSE) score ≤ 26 points [19]), presence of clinical significant morbidity (haematological, renal, endocrine, pulmonary, gastrointestinal, cardiovascular, hepatic, psychiatric, neurological, musculoskeletal or allergic disorders), presence of orthostatic hypotension and use of medication. This study was approved by the Medical Ethics Committee of the Leiden University Medical Center, Leiden, the Netherlands, and was performed according to the principles of the Declaration of Helsinki and the International Conference on Harmonization/Good Clinical Practice (ICH/GCP). All participants gave written informed consent before entry to the study.

### Participant characteristics

Prior to participation, a screening procedure was performed. Medical history was recorded including general questions about smoking, alcohol use, medication use and information on diseases. Anthropometric data included height and body composition measured with a bioelectrical impedance analysis (BIA, InBody 720, Biospace Co., Ltd, Seoul, Korea). Cognitive function was assessed with the MMSE [19]. Orthostatic hypotension was assessed by measuring blood pressure after at least 5 minutes in supine position and 3 minutes after postural change to standing position. Handgrip strength was measured using the Jamar dynamometer handle (Jamar, Sammons Preston Inc, Bolingbrook, IL, USA). Physical functioning was measured with the Short Physical Performance Battery (SPPB) [20]. Walking speed was determined by a 4 meter walking test at normal pace, as part of the SPPB.

### Apparatus

**Fig 1:**
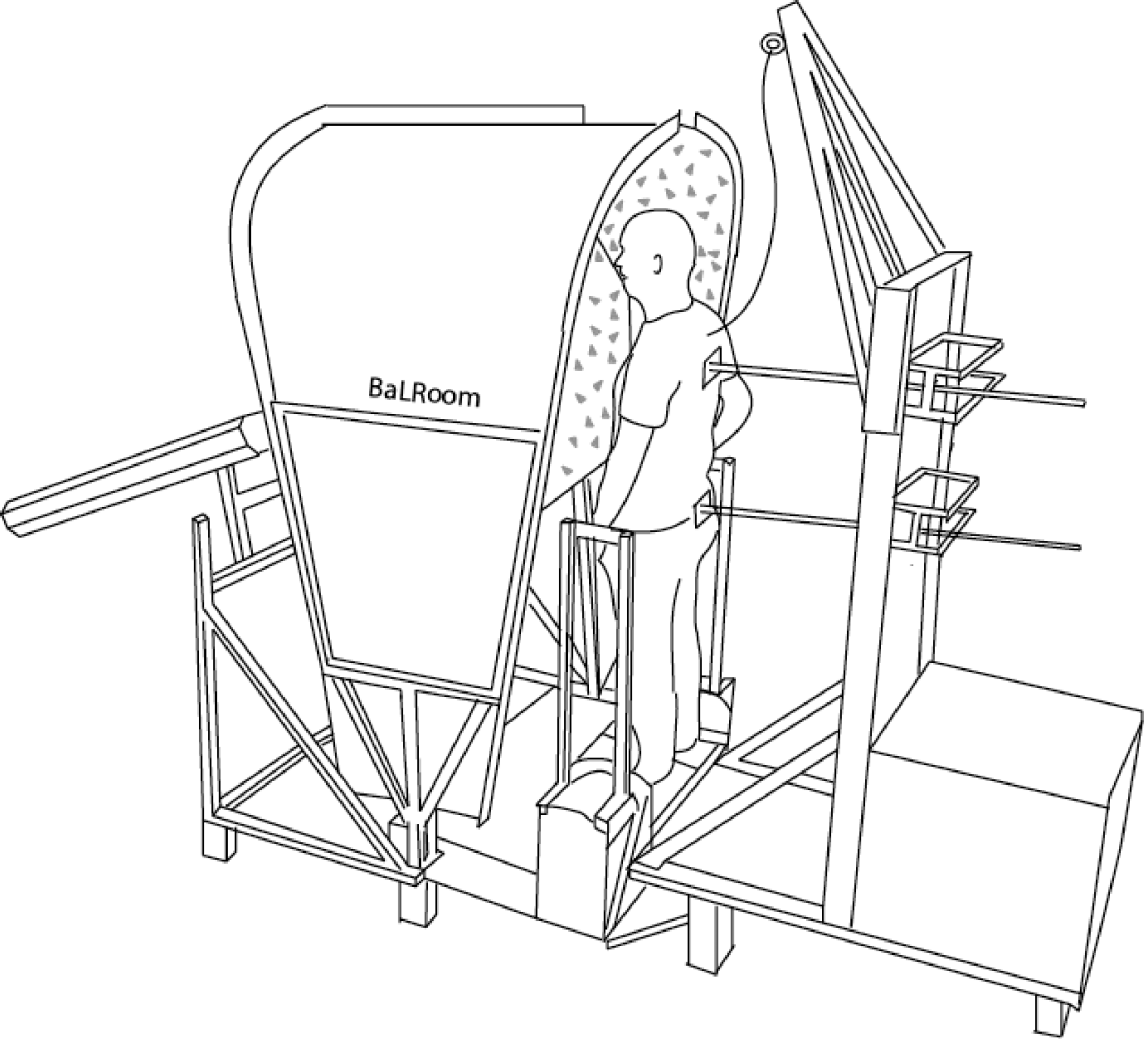
Schematic set up of the Balance test Room consisting of three modules. 1) a visual scene to apply disturbances to the visual system (VS rotation), 2) support surfaces to apply disturbances to the proprioceptive system (SS rotation), and 3) two rods to apply mechanical disturbances by giving pushes and pulls at hip and shoulder level (FH and FS).

Standing balance was assessed using the Balance test Room (BalRoom), a custom-made device applying specifically designed disturbances during stance (Motekforce Link, Culemborg, the Netherlands, and University of Twente, Enschede, the Netherlands) (Fig 1). The BalRoom consists of three separated modules. The first module consists of two support surfaces (SS), which are independently actuated and rotate around the ankles [21]. By rotation of the SS around the ankle axis the proprioceptive information of the ankle is disturbed. The second module is a visual scene (VS) in front of the participant, which rotates around the ankle axes. Rotating the VS around the ankle axis results in a disturbance of the visual information. The third module consists of two rods applying forces at hip and shoulder level (FH and FS, respectively) resulting in movements around the ankle and hip joint. These disturbances are used to investigate the contribution of the ankles and hips and their coupling to standing balance [14].

The body sway was measured in a single plane using a string potentiometer (Celesco SP2-50, Celesco, Chatsworth, CA, United States), which integrates the amplitude of unidirectional body movement transferred through a string attached to the waist of the participant.

### Disturbance signals

**Fig 2:**
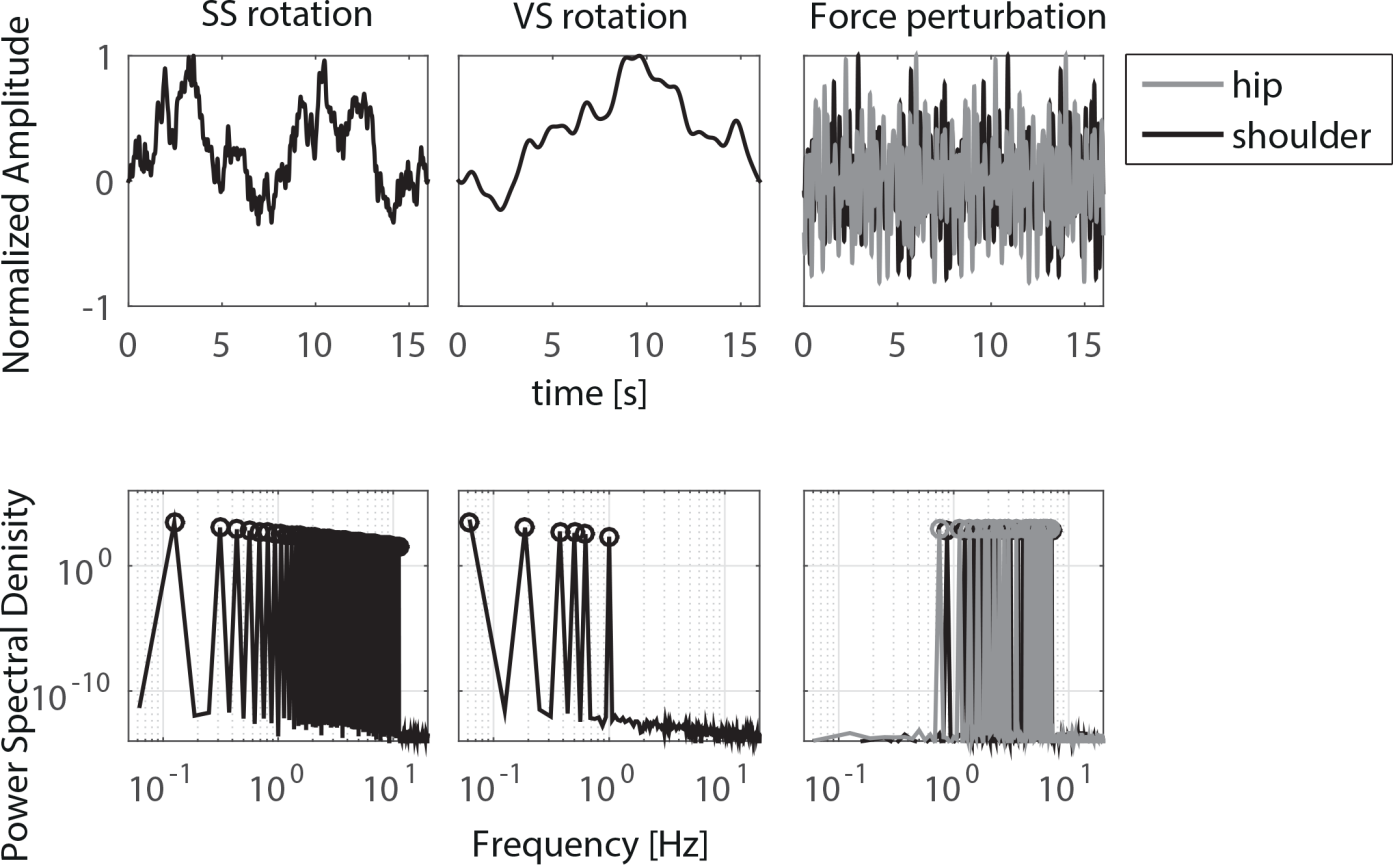
Normalized time signals and frequency spectra of the disturbances of the support surface (SS) rotation, the visual scene (VS) rotation and the rods applying forces at hip and shoulder level (FH and FS, respectively).

All disturbances applied with the BalRoom were multisine signals with a unique combination of frequencies (Fig 2). All excited frequencies were multiples of the frequency 0.0625 Hz resulting in a disturbance period of 16 s. The SS rotated following a continuous position disturbance signal with increasing zero-to-peak amplitude over trials, i.e. 0.02, 0.03 and 0.04 radians, and a flat velocity spectrum with frequencies between 0.125 and 6.9375 Hz. The VS rotated following a continuous position disturbance signal with constant zero-to-peak amplitude of 0.03 radians over trials and a flat velocity spectrum with frequencies between 0.0625 and 1 Hz. The FH and FS disturbances are independent continuous force disturbance signals with constant zero-to-peak amplitude of 30 Newton over trials consisting of frequency contents between 0.75 and 7 Hz. All disturbances were repeated eight times resulting in a total duration of 128 seconds.

### Procedure

During the screening visit for inclusion up to 21 days before the start of the study, each participant had a training session to get familiarized with the BalRoom and with the body sway test. No data were recorded. During the study, the tests were performed during three sessions separated by one week, allowing assessment of intersession variability. Per session the tests were performed twice separated by one hour, allowing assessment of intrasession variability. During all tests the participant wore comfortable flat shoes. During the BalRoom test, the participant was instructed to stand with the arms resting along the body, with both feet in place on the support surfaces. The two sensory (SS and VS) and mechanical (FH and FS) disturbances were applied simultaneously. Each test consisted of three conditions with increasing disturbance amplitude of the SS rotation (i.e. 0.02, 0.03 and 0.04 radians), while the amplitudes of the VS, FH and FS disturbances remained constant. The three conditions were presented in random order. Before recording each condition, the participant was allowed about 10 seconds to get accustomed to the disturbances. Between conditions, the participant was offered ample resting time depending on individual needs. The participant wore a safety harness to prevent falling, which did not constrain movement nor provide support or orientation information.

During the body sway test, the participant was asked to stand still and comfortable with eyes closed for a period of 2 minutes, with the feet approximately 10 cm apart and the hands in a relaxed position along the body.

### Data recording and processing

The actual angles of SS rotation (i.e. motor angles), applied forces at hip and shoulder level (FH and FS forces) and the applied torques to the SS (i.e. motor torques) were available for measurement. Lower and upper body segmental movements were measured in anterior-posterior direction using two draw wire potentiometers (Celesco SP2-50, Celesco, Chatsworth, CA, United States) at a sample frequency of 1000 Hz. The potentiometers were connected to the hip and the shoulders by magnets and straps. The motor angles, segment angles, motor torques and applied FH and FS forces were recorded using a Matlab interface with a sample frequency of 1000 Hz. Data analysis was performed with Matlab (The MathWorks, Natick, MA, United States). The leg and hip angle were calculated using goniometry and using the segment movement of the lower and upper body [22]. The ankle torque was obtained by subtracting the contribution of the mass and inertia of the support surfaces from the recorded motor torques. The hip torque was obtained using the applied FH and FS forces and leg and hip angles using inverse dynamics [22]. The time series were segmented into eight data blocks of 16 seconds (i.e. the period of the disturbance signal).

### Data analysis

To indicate the effect of the disturbances on the ankle torque, hip torque and joint angles, Frequency Response Functions (FRFs) were estimated. The time series of the disturbances, ankle torque, hip torque, leg and hip angle were transformed to the frequency domain. The periodic part of the frequency coefficients was determined by averaging over the data blocks. The Power Spectral Densities (PSD) and Cross Spectral Densities (CSD) were computed to calculate the FRFs [23]. For each disturbance, only the excited frequencies were analyzed.

#### Sensitivity functions

The sensitivity function represents the sensitivity of the body reactions (i.e. joint angles and joint torques) to sensory perturbations. FRFs representing the sensitivity functions of the SS rotation and the VS rotation to the ankle torque, hip torque, leg angle and hip angle were estimated using the indirect approach using Eq 1 [12;23].

$${}^{d}S_{x}\left(f\right)=\Phi_{d,x}\left(f\right)\cdot {\left[\Phi_{d,d}\left(f\right)\right]}^{-1}$$ (1)

In which *Φ*_*d*,*x*_ represents the CSD of the disturbance (*d*) (i.e. SS rotation or VS rotation) and *x*, which represents the ankle torque (*T*_*a*_), hip torque (*T*_*h*_), leg angle (*θ*_*l*_), or hip angle (*θ*_*h*_), and *Φ*_*d*,*d*_ the PSD of the disturbance. This results in 8 FRFs; 1) SS rotation to ankle torque (^*SS*^*S*_*Ta*_), 2) SS rotation to hip torque (^*SS*^*S*_*Th*_), 3) SS rotation to leg angle (^*SS*^*S*_*θl*_), and 4) SS rotation to hip angle (^*SS*^*S*_*θh*_), and 5) to 8) the VS rotation to each torque and angle (^*VS*^*S*_*Ta*_,^*VS*^*S*_*Th*_, ^*VS*^*S*_*θl*_,^*VS*^*S*_*θh*_). Each FRF is represented by a magnitude and phase representing the ratio between the input and output and the relative timing both as function of frequency. The magnitude of the sensitivity function of the ankle and hip torque is normalized to the gravitational stiffness (*mgl*_*CoM*_). The average magnitude on the low frequencies (<0.375Hz and <0.1875Hz, for SS and VS respectively) and the phase on higher frequencies (0.68Hz and 0.375Hz, for SS and VS respectively) are the parameters of interest. Different values of frequencies were used for SS and VS due to differences in frequency content. They represent the sensitivity to the disturbances and the phase lag between the disturbance and the reaction of the body, respectively, resulting in 16 parameters.

#### Neuromuscular controller

The neuromuscular controller is the link between the sensory systems and the muscles, where the sensory information is combined and muscle commands are generated to keep the body in upright position. The FRFs representing the ankle and hip controller and their coupling were estimated using the multi-input-multi-output (MIMO) approach according to the method described by Engelhart et al. (2014) and Eq 2 [14].

$$H_{c}\left(f\right)=-\Phi_{d,T}\left(f\right)\cdot {\left[\Phi_{d,\theta }\left(f\right)\right]}^{-1}$$ (2)

In which *Φ*_*d*,*T*_ and *Φ*_*d*,*θ*_ are the CSD matrices between the external disturbance (*d) (*i.e. FH and FS)) and the corrective ankle and hip torques (*T*) and the leg and hip angles (*θ*) resulting in a two-by-two FRF matrix (*H*_*c*_). This results in 4 FRFs; 1) leg angle to ankle torque (*H*_*θl2Ta*_), 2) leg angle to hip torque (*H*_*θl2Th*_), 3) hip angle to hip torque (*H*_*θh2Th*_), and 4) hip angle to ankle torque (*H*_*θh2Ta*_). The magnitude is normalized to the gravitational stiffness (*mgl*_*CoM*_).The average magnitude on the low frequencies (<1Hz) and the phase on higher frequencies (2.3Hz) are the parameters of interest and represent the normalized effective stiffness and the phase lag between the torques and angles, resulting in 8 parameters [14].

#### Body sway

The body sway (*x*_*BS*_) was measured over 2 minutes during quiet stance with eyes closed. The movement of the body was expressed as millimeters of sway during 2 minutes.

### Statistical analysis

The characteristics of the participants were represented by mean and standard deviation in case of a Gaussian distribution. Else, median and inter quartile range or number and percentage were presented. The parameters obtained with system identification techniques (i.e. sensitivity and phase lag of the sensitivity functions, and normalized effective stiffness and phase lag of the neuromuscular controller) and body sway are given as mean and standard deviation.

Reliability of each parameter was assessed using the G theory in three steps [17]. First, systematic errors were identified using linear mixed models with trial (intrasession), day (intersession) and their interaction as fixed effects and participant intercept as random effect. Because of the number of dependent variables tested, a Bonferroni correction was applied to avoid type I errors. P values below 0.006 were considered statistically significant. The various sources of measurement errors of each parameter were assessed using a random effects repeated measures analysis of variance (ANOVA) including participant, trial, day and their interactions. This resulted in the variance of the participants (*σ*_*p*_^*2*^), the variance of the trials (*σ*_*t*_^*2*^), the variance of the day (*σ*_*d*_^*2*^), the variance of their interactions (*σ*_*pt*_^*2*^, *σ*_*pd*_^*2*^ and *σ*_*td*_^*2*^) and the variance of the residual (*σ*_*ptd*,*e*_^*2*^). All were presented as percentages of the total variance. Negative variance components were set to zero. The actual sources of variance were used to calculate the index of dependability (ID), the standard error of the measurement (SEM) and the minimal detectable change (MDC) using Eq 3 [17;24].

$$\begin{array}{l} \sigma_{\Delta }^{2}=\frac{\sigma_{t}^{2}}{n_{t}}+\frac{\sigma_{d}^{2}}{n_{d}}+\frac{\sigma_{pt}^{2}}{n_{t}}+\frac{\sigma_{pd}^{2}}{n_{d}}+\frac{\sigma_{td}^{2}}{n_{t}n_{d}}+\frac{\sigma_{ptd,e}^{2}}{n_{t}n_{d}}\\ ID=\frac{\sigma_{p}^{2}}{\sigma_{p}^{2}+\sigma_{\Delta }^{2}}\\ SEM=\sqrt{\sigma_{\Delta }^{2}}\\ MDC=1.96*\sqrt{2}*SEM \end{array}$$ (3)

In which, *n*_*t*_ is the number of trials and *n*_*d*_ the number of days.

Comparable with an intraclass correlation coefficient (ICC), the ID ranges between 0 and 1 and can be interpreted as; ID < 0.40 poor reliability, 0.40 < ID < 0.75 moderate reliability, and ID > 0.75 excellent reliability [25]. In this case, the ID represents the reliability for two trials on three days. The SEM indicates the absolute reliability and is represented by an absolute value and a percentage of the overall mean. The MDC shows which effect (e.g. treatment effect) can be detected with the parameters of interest and therefore indicates the clinical relevance. A low SEM and MDC are indicative of a reliable and clinical relevant parameter.

Second, a decision study was performed in which the effect of different measurement protocols on the reliability was investigated. Aforementioned equations show that increasing the number of trials or number of days results in an increase of ID and a decrease of SEM and MDC, i.e. an improvement of reliability. In the decision study, the number of trials was varied between 1 and 40 trials and the number of days between 1 and 3. Per number of days, the number of trials needed to reach an excellent reliability was determined in this group of healthy elderly (ID > 0.75).

Third, a validity study was performed to assess whether differences in the sensitivity functions represented by the sensitivity and phase lag due to increasing disturbance amplitude of the SS rotation could be detected. Previous studies showed an increase in sensitivity to VS rotation [12;26] and a decrease in sensitivity to SS rotation [12;18] due to increasing disturbance amplitude of the SS rotation. Furthermore, no differences in neuromuscular controller were detected with increasing disturbance amplitude [15;18]. A linear mixed model was constructed with disturbance amplitude as fixed effect and participant intercept as random effect. To correct for multiple testing, a Bonferroni correction was applied to avoid type I errors. P values below 0.006 were considered statistically significant.

Statistical analysis was performed with SPSS version 20 (SPSS Inc., Chicago, USA) and Matlab (The MathWorks, Natick, MA, United States). Graphs were made with Matlab (The MathWorks, Natick, MA, United States).

The minimal dataset used for statistical analysis is available from the 3TU database (datacentrum.3tu.nl, DOI: 10.5072/uuid:433acf72-2779-4470-a111-d94c415125b8).

## Results

### Participant characteristics

**Fig 3:**
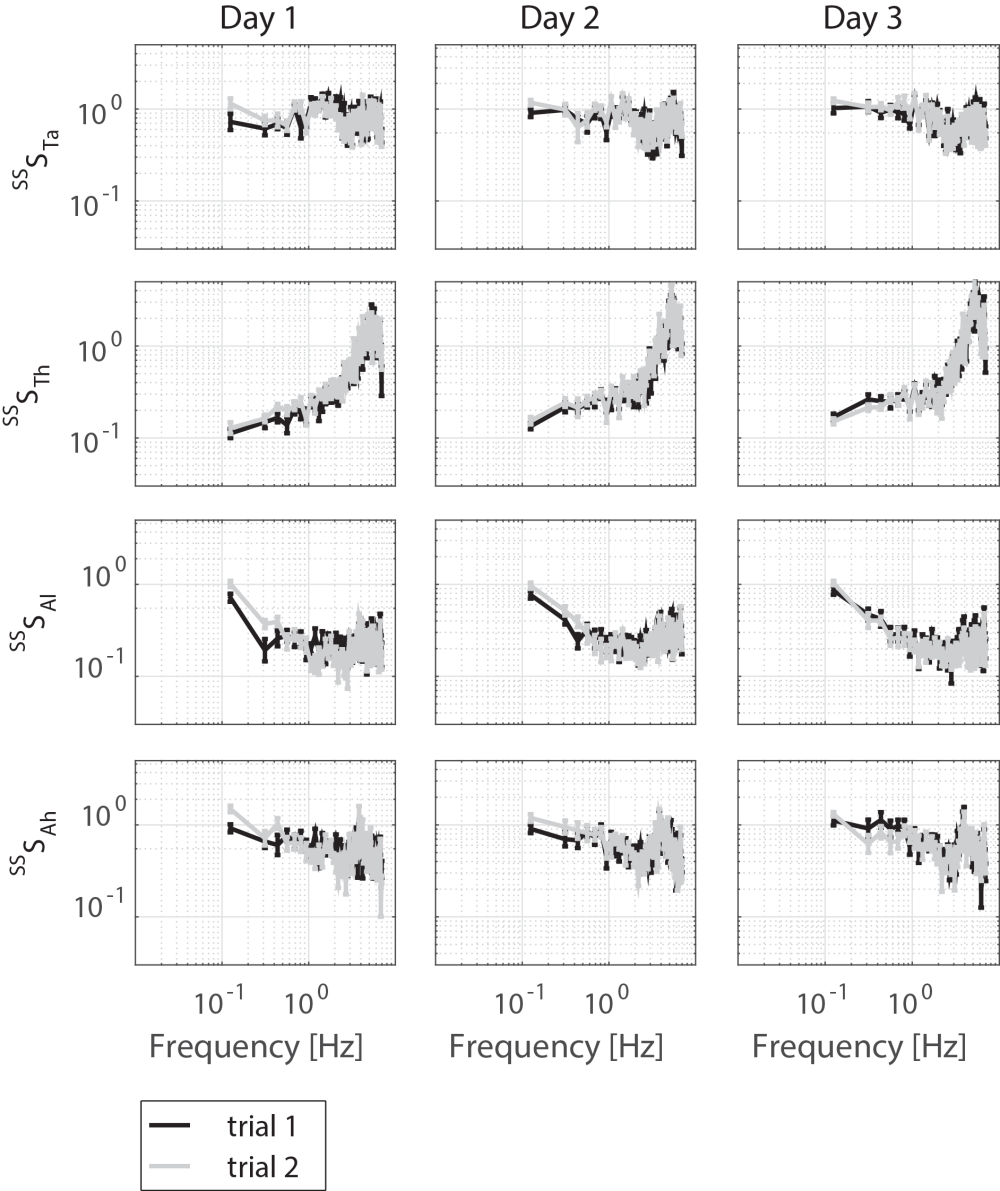
Sensitivity functions (averaged over participants) of the ankle torque (^SS^S_Ta_), hip torque (^SS^S_Th_), leg angle (^SS^S_θl_) and hip angle (^SS^S_θh_) to the rotation of the support surfaces per day per trial are presented by mean and standard error, only magnitude is shown.

**Fig 4:**
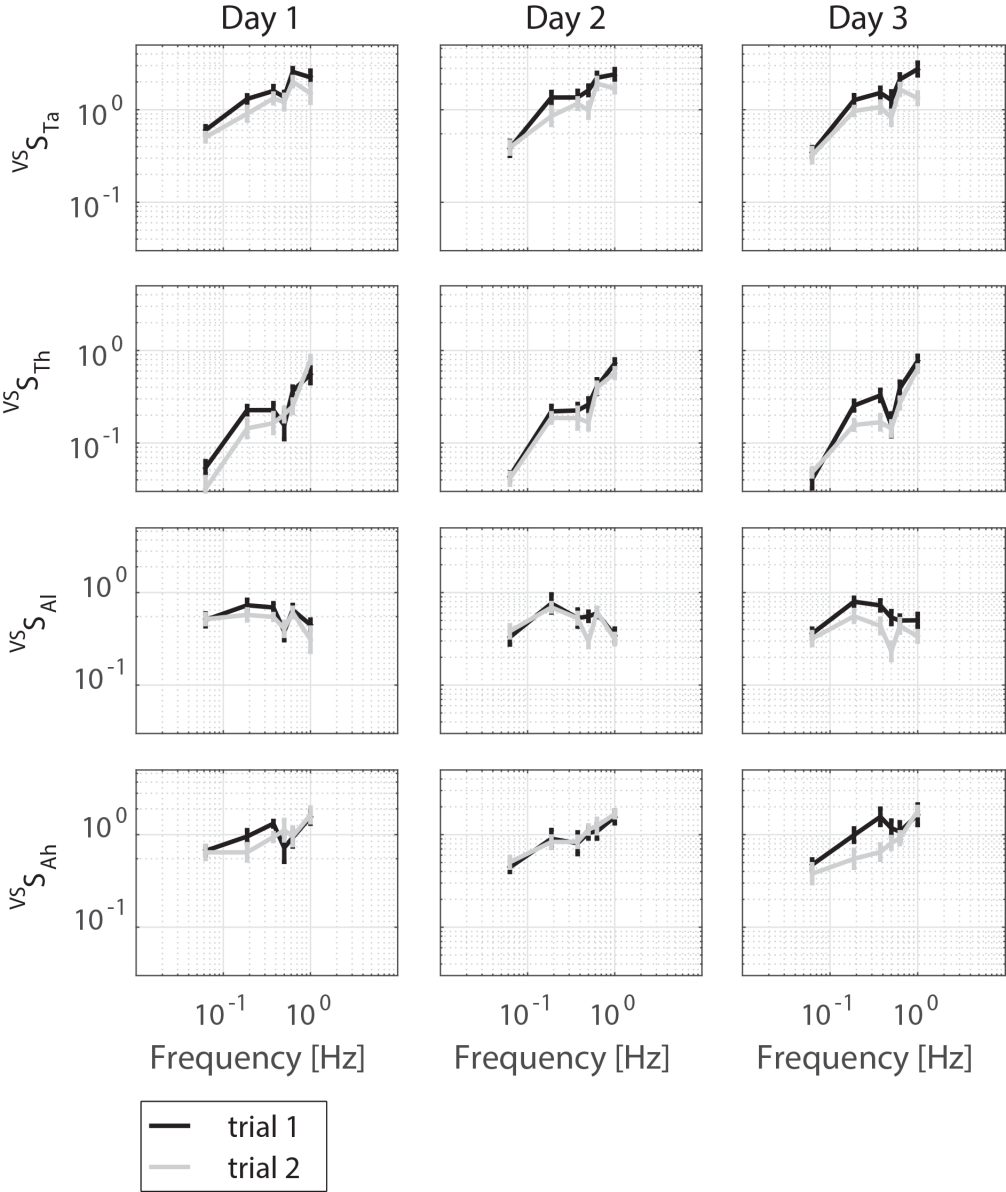
Sensitivity functions (averaged over participants) of the ankle torque (^VS^S_Ta_), hip torque (^VS^S_Th_), leg angle (^VS^S_θl_) and hip angle (^VS^S_θh_) to the rotation of the visual scene per day per trial are presented by mean and standard error, only magnitude is shown.

**Fig 5:**
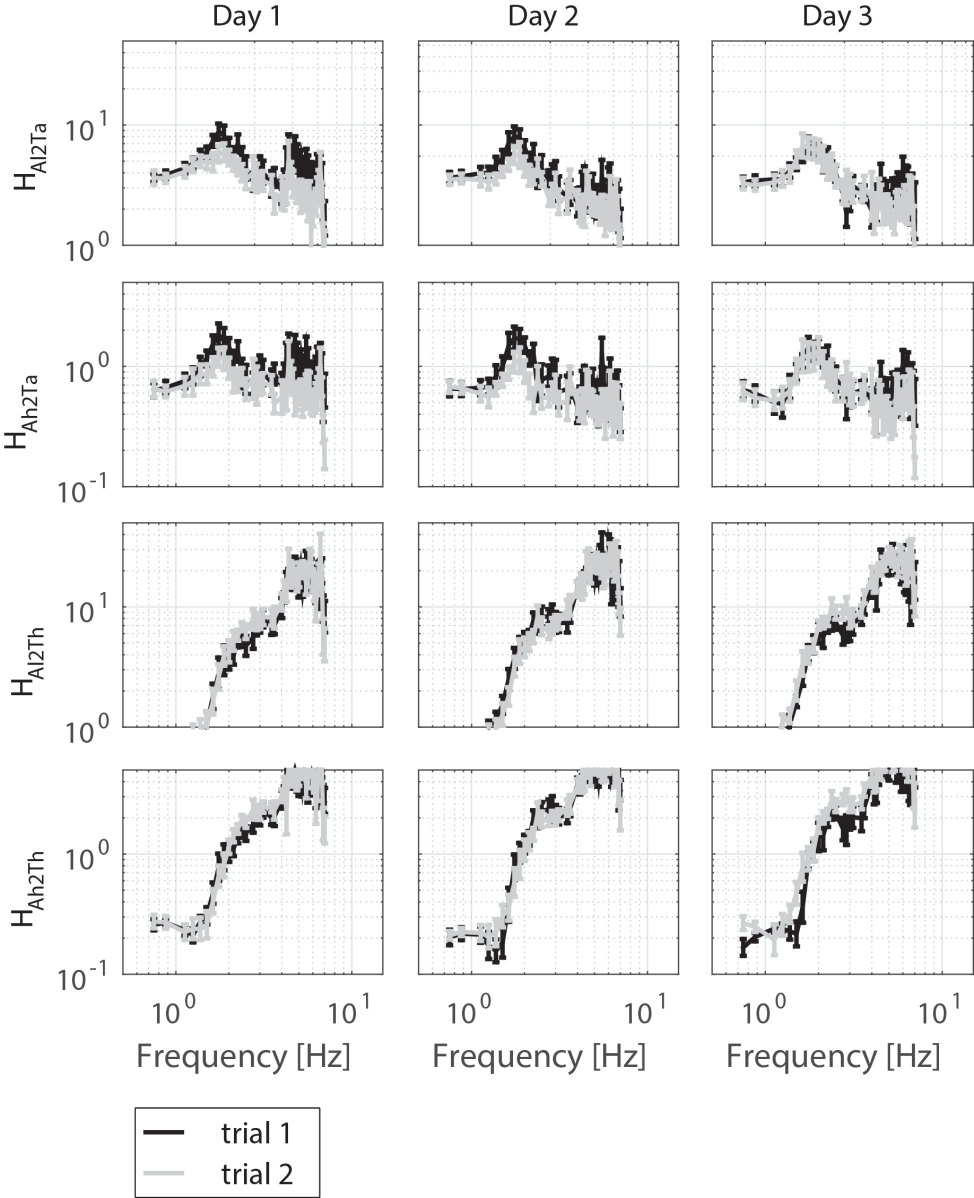
Frequency Response Functions (averaged over participants) of the neuromuscular controller (i.e. H_Ta2θl_, H_Ta2θh_, H_Th2θl_, H_Th2θh_) per day per trial are presented by mean and standard error, only magnitude is shown.

Table 1 presents the characteristics of the healthy old participants. Figs 3, 4 and 5 displays the magnitude of the FRFs of the sensitivity functions of the SS rotations and the VS rotations, and of the neuromuscular controller, respectively.

**Table 1:** Participant characteristics of all participants (n = 12).

	All (n = 12)
Age, years	73.3 (3.4)
Men, n (%)	6 (50)
**Anthropometry**	
Weight, kg	72.2 (9.1)
Height, m	1.70 (0.08)
BMI, kg/m^2^	24.9 (2.4)
**Health characteristics**	
Number of medication, median (IQR)	0 (0–0)
MMSE, points; median (IQR)	29 (28–30)
**Physical function**	
Handgrip strength, kg	34.7 (8.6)
Gait speed, m/s	1.28 (0.16)
SPPB, points; median (IQR)	11 (10–12)

All parameters are presented as mean with standard deviation unless indicated otherwise. BMI: body mass index, MMSE: Mini Mental State Examination, SPPB: Short Physical Performance Battery, IQR: inter quartile range.

### Systematic errors

Table 2 reports the systematic errors obtained with the linear mixed models according to the G theory. No systematic errors were found for the body sway (*x*_*BS*_). The sensitivity functions show both a main effect of trial and day. Overall, the sensitivity of the SS rotation was lower during the first trial compared with the second trial (for ^*SS*^*S*_*θl*_ and ^*SS*^*S*_*Ta*_) and it was lower during the first day compared to the second and third day (for ^*SS*^*S*_*Th*_ and ^*SS*^*S*_*Ta*_). The sensitivity function of the VS rotation shows the opposite result; the sensitivity of the first trial was higher compared with the second trial (for ^*VS*^*S*_*Th*_ and ^*VS*^*S*_*Ta*_). Furthermore, the phase lags of ^*VS*^*S*_*θl*_ was higher in the first trial compared with the second trial. The phase lags did not differ between days.

**Table 2:** Systematic errors of all parameters using linear mixed model with day, trial and their interaction as fixed effect and subject intercept as random effect.

		p-value			Post hoc analyse	
	Mean (SD)	trial (t)	day (d)	t x d	trial	day
***Body sway***						
*x*_*BS*_, mm	330.53 (139.12)	0.18	0.47	0.29	-	-
***Sensitivity functions***						
Sensitivity						
^*SS*^*S*_*θh*_	1.03 (0.39)	0.012	0.26	0.011	-	-
^*SS*^*S*_*θl*_	0.65 (0.17)	**<0.001**	0.024	0.133	Trial 1 < Trial 2	-
^*SS*^*S*_*Ta*_	0.99 (0.27)	**0.002**	**0.001**	0.42	Trial 1 < Trial 2	Day 1 < Day 3
^*SS*^*S*_*Th*_	0.17 (0.05)	0.29	**<0.001**	0.030	-	Day 1 < Day 2 and 3
^*VS*^*S*_*θh*_	0.77 (0.28)	0.018	0.27	0.48	-	-
^*VS*^*S*_*θl*_	0.61 (0.20)	0.044	0.29	0.89	-	-
^*VS*^*S*_*Ta*_	0.86 (0.29)	**0.001**	0.27	0.93	Trial 1 > Trial 2	-
^*VS*^*S*_*Th*_	0.13 (0.06)	**<0.001**	0.72	0.34	Trial 1 > Trial 2	-
Phase lag						
^*SS*^*S*_*θh*_, deg	118.32 (22.73)	0.12	0.54	0.27	-	-
^*SS*^*S*_*θl*_, deg	-94.97 (32.15)	0.23	0.99	0.66	-	-
^*SS*^*S*_*Ta*_, deg	17.29 (15.61)	0.18	0.012	0.028	-	-
^*SS*^*S*_*Th*_, deg	-35.94 (23.67)	0.60	0.41	0.082	-	-
^*VS*^*S*_*θh*_, deg	83.19 (67.21)	0.059	0.11	0.74	-	-
^*VS*^*S*_*θl*_, deg	-97.56 (34.93)	**0.002**	0.13	0.23	Trial 1 > Trial 2	-
^*VS*^*S*_*Ta*_, deg	39.26 (23.80)	0.17	0.033	0.060	-	-
^*VS*^*S*_*Th*_, deg	1.60 (36.14)	0.066	0.76	0.50	-	-
***Neuromuscular controller***						
Normalized effective stiffness						
*H*_*θh2Ta*_	0.71 (0.33)	0.96	0.77	0.95	-	-
*H*_*θh2Th*_	0.25 (0.10)	0.17	0.020	0.18	-	-
*H*_*θl2Ta*_	3.72 (1.29)	0.84	0.19	0.85	-	-
*H*_*θl2Th*_	0.35 (0.24)	0.40	0.21	0.74	-	-
Phase lag						
*H*_*θh2Ta*_, deg	-115.26 (52.78)	0.71	0.15	0.69	-	-
*H*_*θh2Th*_, deg	84.83 (33.62)	0.30	0.042	0.62	-	-
*H*_*θl2Ta*_, deg	-112.71 (38.18)	0.79	0.47	0.81	-	-
*H*_*θl2Th*_, deg	96.10 (38.13)	0.37	0.078	0.74	-	-

Significant differences identified in bold. n.s.: not significant.

The normalized effective stiffness estimated using the FS and FH disturbances showed an effect of the day; one component of the neuromuscular controller (*H*_*θh2Th*_) was higher during the first day compared with the second day. No effect of trial and day was found for the phase lags of all components of the neuromuscular controller.

### Variance components

Table 3 shows the magnitude of the variance components as percentage of the total variance (i.e. the sources of variability) according to the G theory. The variance of the participant in the body sway was 87.3%. The other variance components in the body sway were low varying from 0–7.2%. The median of the variance of the participant (*σ*_*p*_^*2*^) was 17.8% with an interquartile range from 9.9% to 28.9%. The contribution of the trial variance (*σ*_*t*_^*2*^) was 0.9% (median) with an interquartile range from 0.0% to 10.5%. The contribution of the day variance (*σ*_*d*_^*2*^) was 0.4% (median) with an interquartile range from 0.0% to 5.7%.

**Table 3:** Relative magnitude of the variance components obtained with the G-study for all parameters obtained with system identification techniques.

	participant (p), %	trial (t), %	day (d), %	p x t, %	p x d, %	t x d, %	p x t x d, e, %
***Body sway***							
*x*_*BS*_	87.3	0.2	0.0	0.0	4.7	0.5	7.2
***Sensitivity functions***							
Sensitivity							
^*SS*^*S*_*θh*_	26.5	0.0	0.0	5.3	10.9	24.3	33.0
^*SS*^*S*_*θl*_	11.8	17.6	0.0	7.0	11.9	12.6	39.0
^*SS*^*S*_*Ta*_	19.3	10.5	14.4	0.0	7.1	0.7	48.0
^*SS*^*S*_*Th*_	20.4	0.0	15.6	0.2	24.1	10.9	28.8
^*VS*^*S*_*θh*_	28.1	8.7	1.5	0.0	0.0	0.0	61.7
^*VS*^*S*_*θl*_	9.7	13.6	0.0	18.3	8.9	0.0	49.5
^*VS*^*S*_*Ta*_	9.9	23.5	0.9	20.6	15.3	0.0	29.6
^*VS*^*S*_*Th*_	38.8	18.3	0.0	0.0	6.1	2.9	33.9
Phase lag							
^*SS*^*S*_*θh*_	28.9	6.7	0.0	0.2	0.0	2.0	62.1
^*SS*^*S*_*θl*_	10.4	0.0	0.0	13.5	0.0	2.9	73.2
^*SS*^*S*_*Ta*_	16.2	0.0	0.4	5.2	13.5	16.5	48.2
^*SS*^*S*_*Th*_	27.2	0.0	0.0	0.0	9.2	12.0	51.6
^*VS*^*S*_*θh*_	6.7	10.6	9.6	3.1	43.6	0.0	26.4
^*VS*^*S*_*θl*_	0.2	12.6	0.0	14.1	25.6	4.5	42.8
^*VS*^*S*_*Ta*_	42.6	0.0	2.6	0.0	0.0	6.7	48.1
^*VS*^*S*_*Th*_	5.3	2.5	0.0	10.8	19.3	0.0	62.2
***Neuromuscular controller***							
Normalized effective stiffness							
*H*_*θh2Ta*_	48.9	1.1	1.7	0.0	0.0	0.0	48.2
*H*_*θh2Th*_	31.0	0.0	6.3	0.0	1.4	0.9	60.5
*H*_*θl2Ta*_	48.4	0.9	3.4	0.0	0.0	0.0	47.3
*H*_*θl2Th*_	17.1	1.2	5.7	0.0	0.0	0.0	76.0
Phase lag							
*H*_*θh2Ta*_	11.4	0.1	0.0	0.0	24.9	0.4	63.1
*H*_*θh2Th*_	0.0	0.0	9.6	8.3	62.9	2.3	16.8
*H*_*θl2Ta*_	17.8	0.0	0.0	3.5	26.0	0.0	52.6
*H*_*θl2Th*_	0.0	0.0	8.1	5.3	68.0	2.0	16.6
**Median (IQR)**	**17.8 (9.9–28.9)**	**0.9 (0–10.5)**	**0.4 (0–5.7)**	**0.2 (0–7)**	**9.2 (0–24.1)**	**0.9 (0–4.5)**	**48.1 (33–60.5)**

Abbreviations: IQR, Inter Quartile Range.

The error variance related to the interactions between the participant and trial (*σ*_*pt*_^*2*^), between participant and day (*σ*_*pd*_^*2*^) and between trial and day (*σ*_*td*_^*2*^) were low; the median of them were 0.2%, 9.2% and 0.9%, respectively.

The largest proportion of measurement variability was due to the participant variability (*σ*_*p*_^*2*^) and the other interactions combined with the residual error (*σ*_*ptd*,*e*_^*2*^) contributing 48.1% (median) ranging from 7.2% to 76.0%.

### Reliability

Table 4 presents the results of the reliability measures. In this study design, the ID represents the reliability for two trials on three days. The ID of the body sway was 0.97. The ID in 4 out of 25 parameters was higher than 0.75 and in 11 out of 25 parameters ID was between 0.40 and 0.75. The SEM and SEM % were inverse related with the ID. Furthermore, the MDC was lower with increased ID. To reach an ID of 0.75, for the body sway one trial was needed. For 28% (7/25) of the parameters at least seven trials were needed to average over one day to reach an ID of 0.75. Increasing the number of days resulted in less trials needed per day to reach an ID higher than 0.75.

**Table 4:** Reliability statistics of the sensitivity functions and neuromuscular controller.

	ID	SEM	SEM %	MDC	# trials / 1 day >0.75	# trials / 2 days>0.75	# trials / 3 days>0.75
***Body sway***							
*x*_*BS*_	**0.97**	25.01	7.57	69.32	1	1	1
***Sensitivity functions***							
Sensitivity							
^*SS*^*S*_*θh*_	0.63	0.17	22.08	0.47	>40	>40	>40
^*SS*^*S*_*θl*_	0.32	0.09	14.98	0.25	>40	7	4
^*SS*^*S*_*Ta*_	0.48	0.13	14.89	0.35	>40	>40	>40
^*SS*^*S*_*Th*_	0.51	0.02	17.38	0.06	>40	9	4
^*VS*^*S*_*θh*_	0.65	0.13	18.84	0.37	>40	>40	>40
^*VS*^*S*_*θl*_	0.26	0.12	46.56	0.32	7	4	3
^*VS*^*S*_*Ta*_	0.23	0.19	4.98	0.51	>40	>40	>40
^*VS*^*S*_*Th*_	0.69	0.03	7.59	0.07	>40	>40	>40
Phase lag							
^*SS*^*S*_*θh*_, deg	0.67	9.42	7.96	26.10	7	4	3
^*SS*^*S*_*θl*_, deg	0.35	16.34	17.20	45.29	26	15	11
^*SS*^*S*_*Ta*_, deg	0.47	6.81	39.39	18.88	>40	>40	24
^*SS*^*S*_*Th*_, deg	0.66	9.98	27.77	27.67	>40	6	3
^*VS*^*S*_*θh*_, deg	0.19	42.10	50.60	116.68	>40	>40	>40
^*VS*^*S*_*θl*_, deg	0.01	19.68	20.17	54.55	>40	>40	>40
^*VS*^*S*_*Ta*_, deg	**0.81**	8.32	21.18	23.05	4	2	2
^*VS*^*S*_*Th*_, deg	0.18	17.55	1097.14	48.66	>40	>40	>40
***Neuromuscular controller***							
Normalized effective stiffness							
*H*_*θh2Ta*_	**0.84**	0.13	12.21	0.35	3	2	1
*H*_*θh2Th*_	0.71	0.04	5.95	0.11	7	4	3
*H*_*θl2Ta*_	**0.84**	0.48	48.10	1.32	3	2	1
*H*_*θl2Th*_	0.53	0.11	65.82	0.31	14	7	5
Phase lag							
*H*_*θh2Ta*_, deg	0.37	25.57	22.19	70.89	>40	>40	>40
*H*_*θh2Th*_, deg	0.00	19.23	22.66	53.29	>40	>40	>40
*H*_*θl2Ta*_, deg	0.48	17.20	15.26	47.69	>40	>40	>40
*H*_*θl2Th*_, deg	0.00	22.02	22.91	61.03	>40	>40	>40

Abbreviations: ID, Index of Dependability (>0.75 identified in bold); SEM, Standard Error of Measurement; MDC, Minimal Detectable Change.

### Validity

Table 5 presents the results of the validity study. The mean and standard deviation of the parameters of the second trial at the first day are given for each condition. All sensitivities to the SS rotation decreased with increasing disturbance amplitude (p < 0.002). The sensitivities to the VS rotation did not significantly change with increasing disturbance amplitude (p > 0.008). No significant differences were found for the phase lag of the sensitivities to the SS rotation and VS rotation. No significant differences were found between the conditions for the parameters describing the neuromuscular controller.

**Table 5:** Mean and standard deviation of the parameters describing the sensitivity functions and the neuromuscular controller corresponding to three conditions with increasing disturbance amplitude, combined with statistical results.

	0.02 rad	0.03 rad	0.04 rad	p-value
***Sensitivity functions***				
Sensitivity				
^*SS*^*S*_*θh*_	1.13 (0.31)	0.94 (0.41)	0.82 (0.24)	**0.001**
^*SS*^*S*_*θl*_	0.73 (0.14)	0.58 (0.13)	0.48 (0.09)	**<0.001**
^*SS*^*S*_*Ta*_	1.08 (0.21)	0.93 (0.20)		**<0.001**
^*SS*^*S*_*Th*_	0.19 (0.05)	0.18 (0.05)	0.15 (0.03)	**0.001**
^*VS*^*S*_*θh*_	0.73 (0.22)	0.82 (0.28)	0.93 (0.27)	0.059
^*VS*^*S*_*θl*_	0.58 (0.14)	0.63 (0.16)	0.77 (0.28)	0.027
^*VS*^*S*_*Ta*_	0.73 (0.25)	0.90 (0.23)	1.04 (0.39)	0.012
^*VS*^*S*_*Th*_	0.12 (0.04)	0.14 (0.06)	0.17 (0.07)	0.008
Phase lag				
^*SS*^*S*_*θh*_, deg	114.74 (19.76)	114.47 (21.32)	115.66 (19.37)	0.87
^*SS*^*S*_*θl*_, deg	-98.15 (24.46)	-105.43 (20.26)	-92.20 (30.52)	0.63
^*SS*^*S*_*Ta*_, deg	16.34 (11.98)	15.39 (8.90)	22.39 (12.54)	0.086
^*SS*^*S*_*Th*_, deg	-39.11 (10.91)	-31.97 (13.53)	-32.71 (16.57)	0.26
^*VS*^*S*_*θh*_, deg	85.52 (58.65)	88.90 (48.95)	94.19 (42.41)	0.59
^*VS*^*S*_*θl*_, deg	-96.04 (31.73)	-105.29 (44.74)	-102.27 (19.63)	0.65
^*VS*^*S*_*Ta*_, deg	43.44 (25.46)	36.72 (27.95)	40.84 (18.80)	0.76
^*VS*^*S*_*Th*_, deg	7.02 (28.36)	-2.47 (32.19)	-3.31 (25.54)	0.12
***Neuromuscular controller***				
Normalized effective stiffness				
*H*_*θh2Ta*_	0.70 (0.26)	0.80 (0.49)	0.75 (0.28)	0.64
*H*_*θh2Th*_	0.22 (0.06)	0.22 (0.08)	0.23 (0.11)	0.65
*H*_*θl2Ta*_	3.70 (1.21)	3.93 (1.60)	3.93 (1.03)	0.55
*H*_*θl2Th*_	0.32 (0.17)	0.34 (0.14)	0.39 (0.21)	0.37
Phase lag				
*H*_*θh2Ta*_, deg	-89.99 (73.93)	-76.50 (66.69)	-110.96 (58.64)	0.33
*H*_*θh2Th*_, deg	94.36 (36.44)	111.42 (40.04)	107.98 (34.96)	0.26
*H*_*θl2Ta*_, deg	-110.27 (58.82)	-86.77 (37.46)	-108.93 (48.49)	0.94
*H*_*θl2Th*_, deg	106.78 (39.04)	120.37 (35.08)	119.63 (33.69)	0.26

## Discussion

In this study, we assessed the reliability of a comprehensive set of parameters obtained with four disturbances applied simultaneously and (MIMO closed loop) system identification techniques describing standing balance in a group of healthy elderly. Results were obtained by measuring standing balance twice during three days. A distinction was made between systematic and random errors. The results showed a systematic error between the first and second trial measured with the BalRoom on one day, which was not found using the body sway measurements. The reliability ranged from moderate to excellent when averaging the two trials of three days (i.e. averaging six trials). To the best of our knowledge, this is the first study that investigated the reliability of system identification techniques to assess standing balance in healthy elderly.

### Systematic errors

In general, the sensitivity to the SS rotation was lower in the first trial compared with the second trial, while the sensitivity to the VS rotation was higher in the first trial compared to the second trial. These results are confirmed by the variance component of the trial (*σ*_*t*_^*2*^) and day (*σ*_*d*_^*2*^); a high variance component of trial and day indicates a systematic error. Previous studies using system identification techniques also showed a systematic error between the first and second trial or between days. These differences were explained by motor learning, changes in posture or stretching of the joints [27–29]. In contrast, in a previous study no learning effects were found. These results might be due to the practice session all participants performed prior to participation in this study [30].

In our study, the differences between the first trial compared with the second trial (i.e. a lower sensitivity to proprioception and a higher sensitivity to vision during the first trial) could be explained by a difference in strategy used to maintain standing balance or familiarization during the test. According to the sensory reweighting hypothesis, sensory information is weighted based on reliability; the weight of the proprioception increased at the cost of a decrease of the weight of the other sensory information [12]. As the sensitivity to the disturbances represents the contribution of the proprioceptive and visual information, the sensitivity to the SS rotation increases, while the sensitivity to the VS rotation decreases.

The combination of mechanical disturbances with sensory disturbances of the visual and proprioceptive information could have resulted in a longer adaptation time or a redundancy of applied strategies to withstand the disturbances. However, comparable systematic errors within a day were found in healthy elderly (unpublished data) in a previous study using only SS rotation to disturb proprioceptive information [18], which suggests that the longer adaptation time is not due to the combination of multiple disturbances. In contrast, no systematic errors were found in healthy young adults (unpublished data). This is an indication of increased adaptation time in elderly compared with young adults.

When a steady state of standing balance is assessed, a familiarization trial is needed on the same day to overcome the systematic error between trials. Excluding the first trial of each day resulted in less systematic errors between days.

### Reliability

The variance component of the participant (*σ*_*p*_^*2*^) as percentage of the overall variance corresponds to the ICC when both *n*_*t*_ and *n*_*d*_ are equal to one. The reliability of the parameters ranged from poor to moderate. To increase reliability of steady state balance assessment, multiple trials on more than one day have to be performed. The ID values indicate that performing two trials on three days results in a reliability ranging from moderate to excellent, which is needed to discriminate between healthy old individuals. A high residual variance (*σ*_*ptd*,*e*_^*2*^) component indicates that a majority of the measurement error is random or can be attributed to error sources not identified in the study.

In this study, relative low SEM% were found (<20%) in 12 out of 25 parameters, which is comparable with other studies using system identification techniques [27]. A low SEM% indicates that the parameter could detect changes over time within the same participant (e.g. effects of intervention or changes in conditions). However, the SEM values depend on the number of trials performed and on the number of days measured. A high SEM% indicates less accurate parameters; in 6 out of 25 parameters a high SEM% (>30%) was found. Therefore, it must be considered whether these parameters are useful or not in assessing standing balance. The MDC values are in the same order as in a previous study using only SS rotation in healthy elderly (unpublished data) and indicates which change in the parameters can be minimally detected, when comparing groups or within the same participant. It is difficult to interpret the MDC results of new parameters. To get more feeling for this measure and to get more insight in the clinical relevance, it is recommended to assess standing balance using system identification techniques in several groups of elderly with a large variance in impaired balance severity and clinical phenotypes [24].

The results showed that the ID of 4 out of 25 parameters was excellent and the ID of 11 out of 25 parameters was moderate. To reach an excellent reliability for steady state balance assessment in one third of the parameters, at least 7 trials on one day are needed. Most of the parameters do not appear to be reliable in this population, unless a very large number of trials are collected on multiple days. In this study, averaging trials across days seems to be more effective than averaging more trials per day. These results are consistent with the variance component of interaction; the variance component of participant x day (*σ*_*pd*_^*2*^) is much higher than the variance component of participant x trial (*σ*_*pt*_^*2*^). This means that the parameters for each participant were more affected by between day than within day sources of error, relative to the other participants. These results are in accordance with previous studies; Lariviere et al. (2015) showed that one till ten trials were needed to assess an excellent reliability for parameters obtained with system identification techniques [27]. A lower reliability seems to be a general feature of position stabilization task in contrast to tracking tasks [28].

### Validity

The validity study showed that differences could be detected within participants by changing the experimental condition. It was possible to detect changes over conditions using one trial. Increasing the disturbance amplitude of the SS rotation resulted in a decreased sensitivity to the SS rotation. This result was expected according to the sensory reweighting hypothesis, as mentioned before. Our findings are therefore also in line with previous studies investigating sensory reweighting during standing balance using system identification techniques [12;15]. However, we also expected to see an increase in sensitivity to the VS rotation as compensation for the decrease in sensitivity to the SS rotation. The absence of this change might be explained by a third sensory system, i.e. the vestibular system. Less use of proprioceptive information could also be accompanied by more use of the vestibular information. Whether someone increases their use of the visual information or their use of the vestibular information could be different per individual. No changes were found in the neuromuscular controller by increasing the disturbance amplitude of the SS rotation. This is following our expectations, as changes in sensory information does not influence the stiffness and damping of the neuromuscular controller. These results are also in accordance with a previous study, in which we showed that the neuromuscular controller did not change with increasing disturbance amplitude of the SS rotation [15;18].

Furthermore, no changes were found in the phase lag of both the sensitivity functions and the neuromuscular controller with increasing the disturbance amplitude of the SS rotation. According to Peterka (2002) we expected to see a difference in the phase lag of the sensitivity functions to the SS rotation [12]. That we did not find a difference, could be explained by the high MDC values and high SEM% for the phase lags.

### System identification techniques compared to posturography

System identification techniques are a new engineering approach to assess standing balance. In contrast with posturography, a general used technique to assess standing balance, it is possible to detect underlying systems and used strategies in standing balance [6;11]. In this study, we assessed standing balance with both system identification techniques and posturography (i.e. body sway). Compared to system identification techniques, no systematic errors and a higher reliability were found for posturography. To reach an excellent reliability in posturography only 1 trial is needed.

In comparison with our results of system identification techniques, studies investigating the reliability of the Sensory Organization Test (SOT) showed a learning effect in healthy young due to changes in postural strategies or through reweighting of sensory information. Remarkably, this learning effect was only present in more demanding test conditions [31]. Studies investigating the reliability of Center of Pressure (CoP) parameters did not find systematic errors [32;33], which is comparable with our results of the body sway, but in contrast with the system identification techniques results. This could be explained by the influence of used strategies to maintain balance on the parameters. CoP parameters only describe objectively standing balance, while system identification techniques also describe the underlying changes. Therefore, changes in strategies between trials will not be detected by CoP parameters and do not influence the reliability of CoP parameters.

The reliability of the SOT was moderate in noninstitutionalized old adults when 2 sessions of the test were performed 1 week apart. To improve the reliability of the computer-generated scores of the SOT, a modification of the scoring system was recommended [34]. The reliability of CoP parameters depends on the test condition, study design, study population and therapeutic interventions [35]. To reach an excellent reliability of CoP parameters, the duration of the trial must be minimal 90 seconds, must by three to five times repeated and must be measured with eyes closed and on a firm surface [35]. Santos et al. (2007) showed that at least 7 repetitions must be performed to reach an excellent reliability for CoP parameters [36]. This is comparable with our study, in which measurements of approximately two minutes were used to assess standing balance with the BalRoom and must be repeated seven times to reach an excellent reliability. The found relative low SEM% (<20%) are comparable with other studies using CoP parameters [36].

### Clinical recommendations

First, the results indicate that there is a systematic error between the first trial and the second trial. This could be due to changes in used strategies to maintain standing balance and time needed to reach a steady state. Therefore, to assess steady state balance we recommend to perform one familiarization trial on each day. Second, results showed that averaging over days is more effective than averaging within days. However, in clinical practice it is often not feasible to measure on more than one day as it is time-consuming. Furthermore, performing multiple measurements on one day could be hampered by fatigue or boredom of the participant, which has to be taken into account. However, measuring less trials on one day will result in lower reliability.

It is recommended to measure more than 7 trials per day to reach an excellent reliability. However, this is only the case for some of the parameters. 16 out of 25 parameters even require more than 40 trials on one day to reach an excellent reliability. Therefore, we have to take this into account and select which parameters are the most important parameters to assess standing balance and represent the underlying changes in standing balance. Furthermore, more research is needed to answer the question whether changes in the measurement protocol (e.g. including a training session, duration of trials, repetitions of the perturbation signal) will improve reliability or not.

As mentioned before, systematic errors might be due to more time needed for reaching a steady state balance or a redundancy of applied strategies. This implies that parameters obtained with system identification techniques are sensitive for detection of adaptation strategies. Besides steady state balance, adaptation strategy and adaptation time may have clinical meaning and need further exploration. System identification techniques are sensitive tools to assess the duration of adaptation of sensory reweighting [37] in contrast to e.g. CoP measurement.

### Strengths and limitations

The strength of this study is the selection of healthy old participants, resulting in a well phenotyped group. However, this also affects ICC and ID. Low variability within the participants (i.e. a homogeneous population) results in lower ICC and ID values and therefore lower relative reliabilities [38;39]. SEM(%) and MDC are measures of absolute reliability and important measures when interpreting results of repeated measures effects of intervention. In a less healthy population with neurological or balance disorders the variability is likely much higher. This may result in higher reliabilities and therefore lower SEM(%) and MDC values, which indicates more accurate and sensitive parameters. Therefore, before using this technique in another population it is recommended to first test reliability in this population of interest. Another strength of this study is the set up with exactly one week between sessions. A limitation of this study is the relative low number of participants. However, a larger number of participants will result in even less variability within the population due to the homegeneity, which might affect the ID as mentioned before. A larger sample size will therefore not automatically result in better reliability. As in this study only two trials were performed per day, it was not possible to assess the number of trials needed to reach an excellent reliability when omitting the first (familiarization) trial from analysis. Therefore, we could not give recommendations on the number of trials needed to reach an excellent reliability after a training session. Furthermore, we could only predict the number of trials needed to reach an excellent reliability.

## Conclusions

This study investigated the reliability of a comprehensive set of parameters obtained with system identification techniques to assess standing balance in a population of healthy elderly. Systematic errors were present between trials showing sensitivity of parameters obtained with system identification techniques for detection of adaptation strategies. To assess steady state balance a training session is recommended. As only a single trial per day resulted in poor to moderate reliability, it is recommended to perform more trials on separate days. Most of the parameters do not appear reliable unless a very large number of trials are collected across multiple days. Within the present framework, acceptable reliability of steady state balance assessment could be achieved in one third of the parameters by measuring and averaging at least seven trials on the same day.
